# Digital technology-assisted labor support in delivery rooms on maternal pain and anxiety: a systematic review and meta-analysis

**DOI:** 10.3389/fmed.2026.1838637

**Published:** 2026-07-22

**Authors:** Shuijuan Zou, Ying Wang, Yinmei Fu

**Affiliations:** Shaoxing Second Hospital Medical Alliance General Hospital, Shaoxing, Zhejiang, China

**Keywords:** anxiety, artificial intelligence, labor pain, meta-analysis, randomized controlled trial, systematic review, virtual reality

## Abstract

**Background:**

Labor pain and anxiety are important factors affecting maternal childbirth experience. Digital technology-assisted labor support, such as virtual reality (VR) analgesia and artificial intelligence (AI)-assisted fetal heart rate monitoring interpretation, has gained increasing attention as non-pharmacological interventions in recent years, but quantitative synthesis of randomized controlled trial evidence remains limited. We aimed to systematically evaluate the effects of digital technology-assisted labor support in delivery rooms on maternal pain and anxiety.

**Methods:**

PubMed, Embase, the Cochrane Central Register of Controlled Trials, Web of Science were systematically searched from inception to March 2026. Randomized controlled trials (RCTs) comparing digital technology-assisted labor support (VR or AI-assisted systems) with routine care on maternal outcomes were included. Two reviewers independently performed study selection, data extraction, and quality assessment using the Cochrane ROB2.0 tool. Meta-analysis was conducted using RevMan 5.4 software, calculating standardized mean differences (SMD) with 95% confidence intervals (CI).

**Results:**

Six RCTs involving 492 parturients were included. Meta-analysis of four studies (362 parturients) showed that digital technology-assisted labor support significantly reduced maternal pain intensity (SMD = −1.49, 95% CI: −2.55 to −0.42, *p* = 0.006, *I*^2^ = 95%) and anxiety levels (SMD = −2.87, 95% CI: −4.37 to −1.36, *p* = 0.0002, *I*^2^ = 97%) compared with routine care. Descriptive findings from two studies not included in the meta-analysis (within-group pre-post comparison and crossover design) were consistent with these results. The overall risk of bias of included studies was moderate.

**Conclusion:**

Current evidence suggests that digital technology-assisted labor support in delivery rooms, particularly immersive virtual reality interventions, can significantly reduce maternal pain intensity and anxiety levels. Due to the limited number of included studies and high heterogeneity, these findings should be interpreted with caution, and more high-quality, large-sample RCTs are needed for further validation.

**Systematic review registration:**

Systematic Reviews (PROSPERO) (Registration number: CRD420261336637).

## Introduction

Childbirth is a unique and profound physiological experience in a woman’s life, yet it is also widely regarded as one of the most intense forms of pain that most women will ever encounter (1, 2). Labor pain involves not only physiological dimensions but also significant psychological stress, including anxiety, fear, and uncertainty (2, 3). If poorly managed, these negative emotions can impair the childbirth experience, heighten the risk of postpartum depression, and even disrupt mother-infant interaction and breastfeeding initiation (4, 5). Therefore, optimizing labor pain management and alleviating maternal anxiety, while ensuring maternal and neonatal safety, have become central objectives of modern obstetric care (6, 7).

Traditionally, labor analgesia has relied primarily on pharmacological interventions, among which neuraxial anesthesia (e.g., epidural analgesia) is widely regarded as the “gold standard” for relieving labor pain (8–10). However, pharmacological analgesia is not suitable for all parturients and may be associated with certain side effects, such as motor blockade, hypotension, urinary retention, prolonged labor, and maternal fever (11, 12). Furthermore, many healthy pregnant women who wish to minimize medical interventions prefer non-pharmacological methods to cope with labor pain, aiming to maintain alertness and mobility while achieving a more natural childbirth experience (10, 13). This demand has driven the development of non-pharmacological analgesic techniques, including hydrotherapy, massage, acupuncture, yoga, breathing techniques, and transcutaneous electrical nerve stimulation (TENS) (14–16).

In recent years, the rapid advancement of digital technologies has opened new possibilities in obstetric care. Among these, virtual reality (VR), as an emerging immersive technology, exerts an analgesic effect by providing engaging audiovisual environments that distract parturients from pain signals (17, 18). Its theoretical foundation is the “distraction hypothesis”: when the brain is occupied by a flood of multisensory information from the virtual environment, its capacity to process pain signals is correspondingly reduced (17). Simultaneously, artificial intelligence (AI) technology is increasingly being applied in obstetrics, particularly in the interpretation of fetal heart rate monitoring. AI-assisted fetal heart rate monitoring systems can analyze complex fetal heart rate data in real-time, assist clinical decision-making, and aim to identify fetal distress early for timely intervention, thereby alleviating maternal anxiety related to uncertainty about fetal well-being (19).

Although several primary studies have explored the effects of VR and AI-assisted systems during labor, the findings remain inconsistent, and sample sizes are generally small. For instance, some randomized controlled trials have shown that VR interventions can significantly reduce maternal pain scores and anxiety levels (20, 21), while other studies have observed only minor improvements or no significant differences (22). To date, there is a lack of systematic integration and quantitative evaluation of the effectiveness of these digital technology interventions.

While several qualitative reviews have addressed virtual reality in obstetric settings, a quantitative meta-analysis focusing specifically on maternal pain and anxiety outcomes across diverse digital technology-assisted labor support interventions has not yet been performed. Therefore, this systematic review and meta-analysis aims to systematically evaluate the effects of digital technology-assisted labor support in delivery rooms on maternal pain intensity, anxiety levels, and delivery outcomes. By synthesizing evidence from existing randomized controlled trials, we hope to provide an evidence-based foundation for obstetric clinical practice and identify directions for the future development and application of digital technologies in perinatal care.

## Methods

### Study design

This systematic review and meta-analysis was conducted and reported in accordance with the Preferred Reporting Items for Systematic Reviews and Meta-Analyses (PRISMA) guidelines (23). The study protocol was registered with the International Prospective Register of Systematic Reviews (PROSPERO) (Registration number: CRD420261336637). As this study involved secondary analysis of published literature, ethical approval was not required.

### Search strategy

We systematically searched the following electronic databases: PubMed, Embase, the Cochrane Central Register of Controlled Trials (CENTRAL) and Web of Science. The search period covered from the inception of each database to March 2026. The search strategy combined Medical Subject Headings (MeSH) terms and free-text keywords, including: “virtual reality,” “VR,” “artificial intelligence,” “AI,” “machine learning,” “fetal monitoring,” “labor pain,” “anxiety,” and “randomized controlled trial.” The detailed search strategy for PubMed is provided in Appendix 1. Additionally, we manually screened the reference lists of all included studies to identify potentially eligible studies.

### Inclusion and exclusion criteria

The inclusion criteria were defined according to the PICOS framework: (1) Participants: Full-term pregnant women with singleton pregnancies, planning vaginal delivery, aged ≥18 years; (2) Interventions: The experimental group received digital technology-assisted labor support, including immersive virtual reality analgesia or AI-assisted fetal heart rate monitoring interpretation; (3) Comparisons: The control group received routine obstetric care, placebo interventions, or other non-digital technology interventions; (4) Outcomes: Primary outcomes were maternal pain intensity (measured by Visual Analogue Scale [VAS] or Numeric Rating Scale [NRS]) and anxiety levels (measured by State Anxiety Inventory [S-AI], Perinatal Anxiety Screening Scale [PASS], or Edinburgh Postnatal Depression Scale [EPDS]); Secondary outcomes included duration of labor stages, mode of delivery, neonatal Apgar scores, and maternal satisfaction; (5) Study design: Randomized controlled trials (RCTs). Exclusion criteria included: non-randomized controlled studies, observational studies, case reports, reviews, conference abstracts; duplicate publications; studies from which valid data could not be extracted; studies not published in English or Chinese.

### Study selection and data extraction

Two reviewers independently performed study selection. Firstly, obviously irrelevant studies were excluded by screening titles and abstracts. Subsequently, full texts of the remaining articles were reviewed to determine final inclusion. Any disagreements were resolved through discussion or by consulting a third reviewer. Data extraction was also independently conducted by two reviewers using a standardized data extraction form. The extracted information included: first author, publication year, country, sample size, maternal characteristics (age, gestational week, parity), intervention details (VR content, duration, frequency; AI system type), control conditions, outcome measures, and time points of outcome assessment. For missing data, we attempted to contact the corresponding authors via email.

### Quality assessment

The risk of bias of included studies was assessed using the Cochrane Collaboration’s Risk of Bias 2.0 (ROB2.0) tool for randomized controlled trials (24). This tool evaluates bias across five domains: bias arising from the randomization process, bias due to deviations from intended interventions, bias due to missing outcome data, bias in measurement of the outcome, and bias in selection of the reported result. Each domain and the overall risk of bias were judged as “low risk,” “some concerns,” or “high risk.” Two reviewers independently performed the assessment, and disagreements were resolved through consensus. The risk of bias assessment results were presented graphically.

### Statistical analysis

All meta-analyses were performed using RevMan 5.4 software (Cochrane Collaboration). For continuous outcomes (e.g., pain scores, anxiety scores), the Standardized Mean Difference (SMD) with 95% Confidence Interval (CI) was calculated. For dichotomous outcomes (e.g., cesarean section rate), the Relative Risk (RR) with 95% CI was calculated. Heterogeneity across included studies was assessed using the *I*^2^ statistic and Cochran’s *Q* test. If *I*^2^ < 50% and the *Q*-test *p* > 0.10, indicating low heterogeneity, a fixed-effects model was applied; otherwise, a random-effects model was used, and subgroup analyses or sensitivity analyses were conducted to explore potential sources of heterogeneity. If a sufficient number of studies (≥10) were included, publication bias was assessed using funnel plots and Egger’s test. Subgroup analyses were planned based on intervention type (VR vs. AI-assisted systems), parity (primiparous vs. multiparous women), and timing of intervention (latent phase vs. active phase). However, due to the limited number of included studies (only one study involved an AI-assisted system), subgroup analyses by intervention type were not statistically meaningful and were therefore not performed. Instead, potential sources of heterogeneity are explored in the Discussion. Sensitivity analyses were performed by sequentially omitting individual studies to evaluate the robustness of the pooled results. In particular, given the conceptual differences between the intelligent management system evaluated and the VR-based interventions in other studies, we conducted a post-hoc sensitivity analysis to assess its influence on the overall effect estimates. A *p*-value <0.05 was considered statistically significant.

Due to methodological heterogeneity in study design, two studies (Carus et al. (20) and Frey et al. (25)) were not included in the primary meta-analysis. Carus 2022 reported only within-group pre-post comparisons, while Frey 2018 employed a crossover design reporting paired differences. The results of these two studies were analyzed descriptively and integrated with the meta-analysis findings in the Discussion section.

## Results

### Study selection

**Figure 1:**
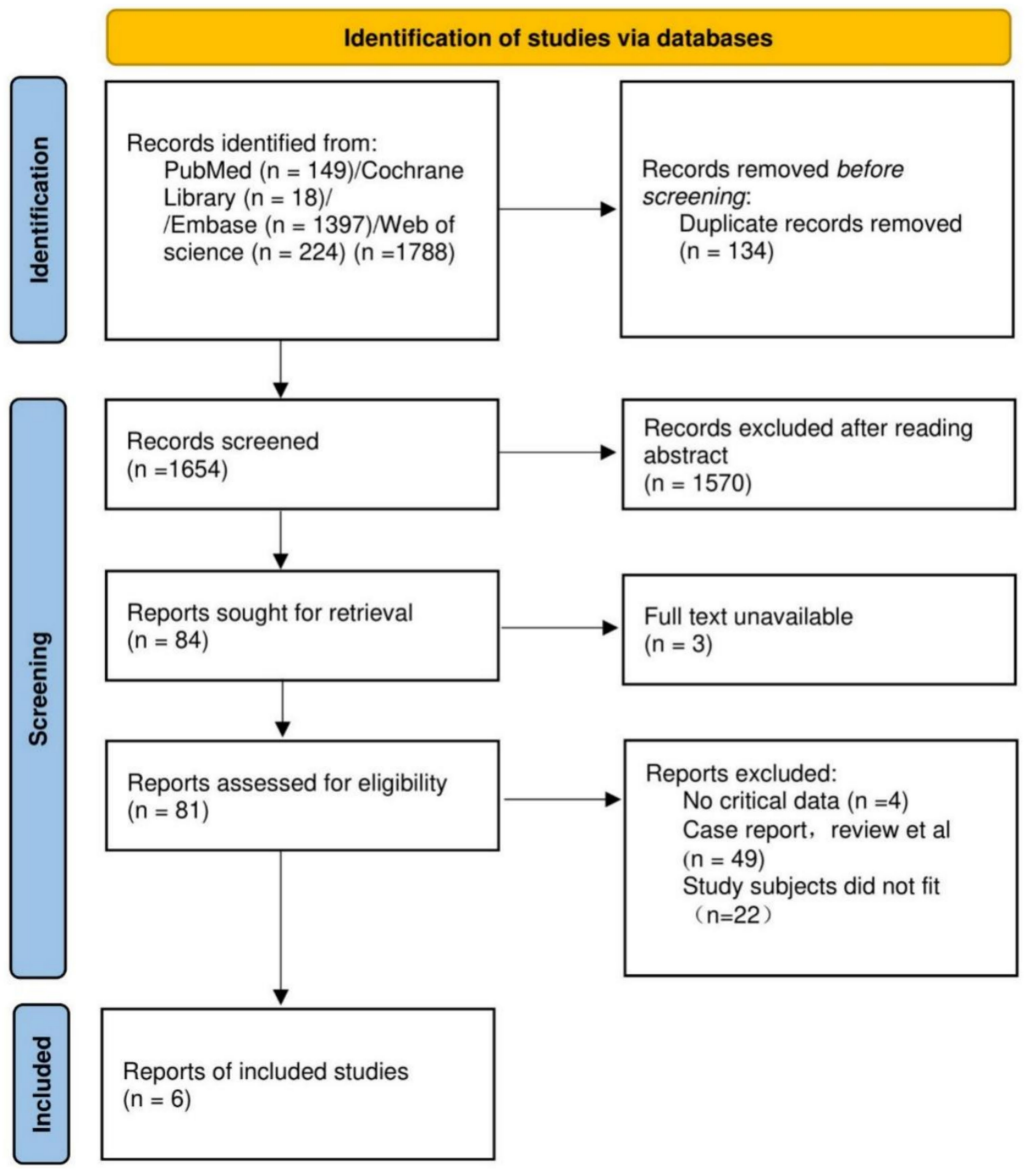
PRISMA flow diagram of study selection.

A total of 1788 records were identified through database searching. After removing duplicates and screening titles and abstracts, 84 full-text articles were assessed for eligibility. Finally, 6 randomized controlled trials (20, 21, 25–28) were included in the systematic review, of which 4 studies (21, 26–28) reporting complete between-group data were included in the meta-analysis, and 2 studies (20, 25) with methodological heterogeneity (within-group pre-post comparison and crossover design, respectively) were analyzed descriptively. The study selection process is presented in Figure 1.

### Study characteristics

The six included studies were published between 2018 and 2023, conducted in Turkey, China, Iran, and the United States. Sample sizes ranged from 27 to 130, comprising a total of 492 parturients. All participants were full-term singleton pregnant women planning vaginal delivery, who were primiparous or of low parity. Interventions included immersive virtual reality with nature scenes, VR games, 4D fetal ultrasound images, and an intelligent delivery room management system. All control groups received routine obstetric care. The baseline characteristics of included studies are summarized in Table 1.

**Table 1:** Characteristics of included studies.

Study (year)	Country	Sample size	Maternal characteristics	Intervention	Control	Outcomes
Carus et al. 2022 (20)	Turkey	*N* = 42 (VR = 21, Control = 21)	Age: 31.0 ± 2.6 (VR), 31.8 ± 3.6 (Control); Gestational week: 39.5 ± 0.6 (VR), 39.0 ± 1.0 (Control); Primiparous: 86% (VR), 81% (Control)	Immersive VR (Oculus Quest) with nature scenes (Nature Treks) for 20 min at 3 cm and 6–7 cm cervical dilation	Routine care	Primary: patient satisfaction (VR satisfaction survey, overall childbirth experience NRS); Secondary: pain (Wong-Baker Faces), anxiety (BAI), depression (BDI)
Ebrahimian et al. 2022 (21)	Iran	*N* = 93 (VR = 31, Chewing gum = 31, Control = 31)	Age: 24.23 ± 4.44 years (mean); Gestational week: 39.31 weeks; Primiparous: 65.6%	VR group: Samsung Gear VR with 360° nature videos for 20 min at 4–5 cm and 7–8 cm dilation; Chewing gum group: sugar-free mint gum chewed for 20 min at same time points	Routine care	Pain (VAS), anxiety (Spielberger State Anxiety Inventory)
Frey et al. 2019 (25)	United States	*N* = 27 (crossover design)	Age: 27.9 ± 5.6 years; Gestational week ≥32 weeks; All primiparous; Unmedicated labor	Samsung GearVR with Ocean Rift simulation (manatees, underwater sounds, relaxing music) for 10 min or 3 contractions	No VR (routine care) in crossover design; each woman served as own control	Pain (sensory, affective, cognitive NRS), anxiety, nausea, satisfaction
Li et al. 2022 (26)	China	*N* = 100 (Observation = 50, Control = 50)	Age: 24.68 ± 4.21 (Control), 25.10 ± 4.22 (Observation); Gestational week: 39.64 ± 0.78 (Control), 39.88 ± 0.72 (Observation); All primiparous	Intelligent delivery room management system (real-time monitoring of analgesia pump and maternal parameters, mobile viewing, family-friendly environment, multidisciplinary collaboration) throughout labor	Routine obstetric care (psychological support, breastfeeding guidance)	Duration of labor stages, pain (NRS), neonatal Apgar, cesarean section rate, anxiety (S-AI), depression (EPDS)
Akin et al. 2021 (27)	Turkey	*N* = 100 (Intervention = 50, Control = 50)	Age: 27.23 ± 3.10 years; All primiparous; Gestational week: 38–41 weeks	4D ultrasound images of fetus recorded at 28 weeks; shown via VR Box 3D headset during labor (mean viewing time 14.18 min)	Routine care	Pain (VAS at 4 cm and 9 cm dilation), supportive care perception (POBS), perinatal anxiety (PASS)
Mohammadi et al. 2023 (28)	Iran	*N* = 130 (VR = 65, Control = 65)	Age: 18–42 years; Gestational week: 37–41 weeks; All primiparous	Samsung Gear VR headset with a game simulating sea shore (boat floating, water sound) starting at 4 cm dilation for ≥20 min until end of first stage	Routine care	Pain (VAS at 4, 6, 8, 10 cm dilation), fear of childbirth (Harman questionnaire)

VR, virtual reality; NRS, numeric rating scale; VAS, visual analogue scale; BAI, Beck Anxiety Inventory; BDI, Beck Depression Inventory; S-AI, State Anxiety Inventory; EPDS, Edinburgh Postnatal Depression Scale; POBS, Perception of Supportive Care Given During Labor Scale; PASS, Perinatal Anxiety Screening Scale.

### Risk of bias assessment

**Figure 2:**
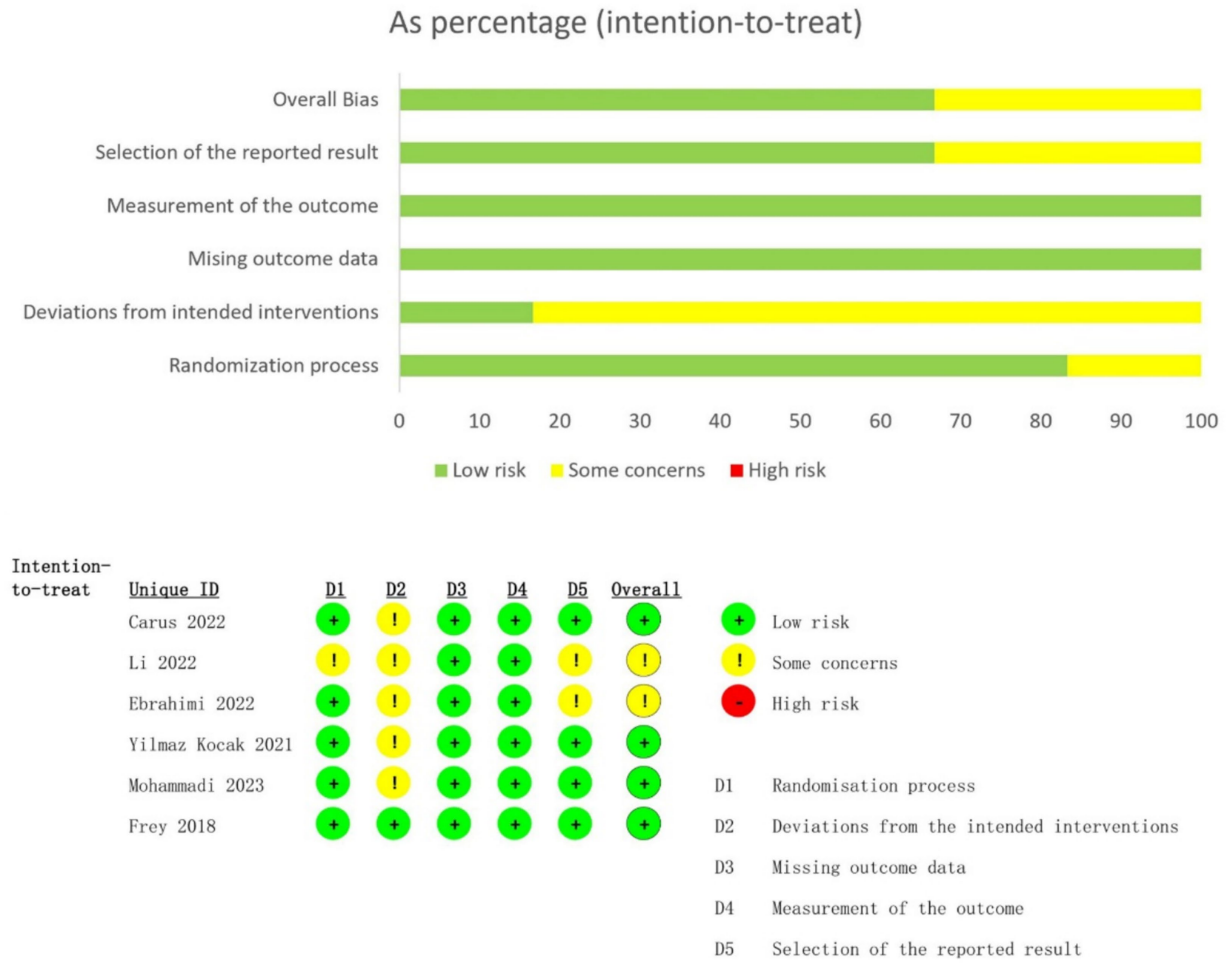
Risk of bias assessment.

The risk of bias of included studies was assessed using the ROB2.0 tool, with detailed results presented in Figure 2. Overall, the included studies had moderate risk of bias. Across the five ROB2 domains, the most common concerns arose from the randomization process (three studies lacked detailed allocation concealment descriptions) and deviations from intended interventions (blinding of participants was not feasible due to the nature of VR interventions, introducing potential performance bias). Missing outcome data was low risk in all studies. Bias in outcome measurement was judged as some concerns for subjective outcomes (pain, anxiety) due to lack of blinding of outcome assessors in most studies. Bias in selection of reported result was low in studies with preregistered protocols (Ebrahimian et al. (21), Mohammadi et al. (28)), but some concerns existed for studies without trial registration (Carus et al. (20), Frey et al. (25), Akin et al. (27), Li et al. (26)).

### Meta-analysis results

#### Maternal pain intensity

**Figure 3:**
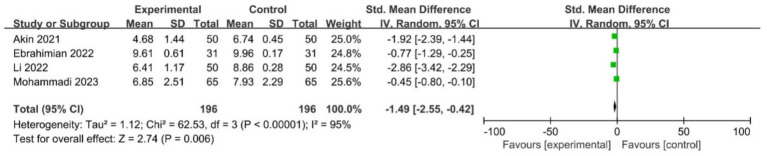
Forest plot of pain intensity.

Four studies (21, 26–28) reported pain intensity, comprising 362 parturients. Due to significant heterogeneity across studies (*I*^2^ = 95%, *p* < 0.00001), a random-effects model was applied. Meta-analysis showed that digital technology-assisted labor support significantly reduced maternal pain intensity compared with routine care, with a pooled standardized mean difference (SMD) of −1.49 (95% confidence interval [CI]: −2.55 to −0.42), and the overall effect test showed Z = 2.74 (*p* = 0.006) (Figure 3). This indicates that pain scores in the intervention group were on average 1.49 standard deviations lower than those in the control group, reaching statistical significance.

#### Anxiety level

**Figure 4:**
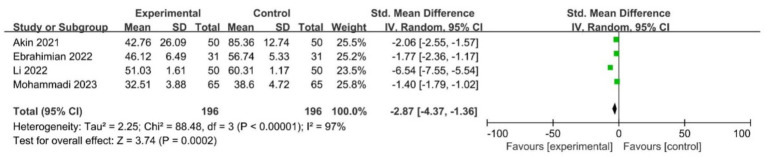
Forest plot of anxiety levels.

Four studies (21, 26–28) reported anxiety or fear of childbirth, comprising 362 parturients. Due to significant heterogeneity across studies (*I*^2^ = 97%, *p* < 0.00001), a random-effects model was applied. Meta-analysis showed that digital technology-assisted labor support significantly reduced maternal anxiety levels compared with routine care, with a pooled SMD of −2.87 (95% CI: −4.37 to −1.36), and the overall effect test showed *Z* = 3.74 (*p* = 0.0002) (Figure 4). This indicates that anxiety scores in the intervention group were on average 2.87 standard deviations lower than those in the control group, reaching high statistical significance.

### Heterogeneity and sensitivity analysis

Both meta-analyses for pain intensity and anxiety showed high heterogeneity (*I*^2^ > 90%). Sensitivity analysis by sequentially omitting each study showed that the pooled effect sizes remained within the range of −1.10 to −1.82 for pain and −2.21 to −3.24 for anxiety, indicating that the results were robust. In particular, given the conceptual differences between the intelligent management system evaluated and the VR-based interventions in other studies, we conducted a post-hoc sensitivity analysis excluding Li et al. (26) to assess its influence on the overall effect estimates. For pain intensity, the updated pooled SMD was −1.04 (95% CI: −1,93 to −0.15), with *I*^2^ = 92%; for anxiety levels, the updated pooled SMD was −1.72 (95% CI: −2.13 to −1.30), with *I*^2^ = 55%. These results further confirm the robustness of our main findings.

### GRADE certainty of evidence

We assessed the certainty of evidence for the two primary outcomes (pain intensity and anxiety level) using the GRADE approach. For pain intensity, the certainty of evidence was very low, downgraded due to very serious inconsistency (*I*^2^ = 95%), serious imprecision (limited sample size of 362 parturients and wide 95% confidence intervals ranging from −2.55 to −0.42), and serious risk of bias (lack of blinding and absence of trial registration in some studies). For anxiety level, the certainty of evidence was also very low, downgraded for the same reasons: very serious inconsistency (*I*^2^ = 97%), serious imprecision (wide confidence interval from −4.37 to −1.36), and serious risk of bias. These very low certainty ratings indicate that our effect estimates are highly uncertain and that future research is very likely to change the estimates.

### Descriptive analysis

Carus et al. (20) reported pre-post pain changes in 21 parturients receiving VR intervention. Pain scores significantly decreased from 2.6 ± 1.2 to 2.0 ± 1.3 (*p* < 0.01) after VR use in early labor. Additionally, overall childbirth satisfaction was significantly higher in the VR group than controls (8.8 ± 1.1 vs. 7.9 ± 1.6, *p* = 0.04), and 95% of women expressed willingness to use VR in future labor.

Frey et al. (25) employed a crossover design in 27 unmedicated laboring women. Compared with the no-VR condition, VR significantly reduced sensory pain (difference −1.5, 95% CI: −2.2 to −0.8), affective pain (difference −2.5, 95% CI −3.3 to −1.6), cognitive pain (difference −3.1, 95% CI −3.8 to −2.4), and anxiety (difference −1.5, 95% CI −2.3 to −0.7). Eighty-two percent of participants reported enjoying the VR experience, and no adverse events were reported.

These descriptive findings are consistent with the direction of the meta-analysis results, further supporting the beneficial effects of digital technology-assisted labor support on pain and anxiety reduction.

### Publication bias

Due to the limited number of studies included in the meta-analysis (*n* = 4), funnel plot analysis and Egger’s test for publication bias were not performed.

## Discussion

To our knowledge, this is the first meta-analysis to quantitatively synthesize the effects of digital technology-assisted labor support—including immersive virtual reality, VR-based fetal visualization, and AI-assisted systems—on maternal pain and anxiety in delivery rooms, based on pooled data from randomized controlled trials. Although previous systematic reviews have examined VR interventions in obstetrics, existing meta-analyses have focused on broad labor parameters or delivery outcomes (29, 30), while qualitative and integrative reviews (31, 32) have described patient experiences without providing quantitative pooled estimates of pain and anxiety. A recent large-scale meta-analysis by Teh et al. (33) demonstrated the effectiveness of VR for pain and anxiety reduction during labour (pain SMD −0.93, 11 studies; anxiety SMD −1.13, 8 studies); however, their review was limited to VR-based interventions and did not include AI/system-based labor support or VR-based fetal visualization, which are unique components of our broader “digital technology” framework. Meta-analysis of four randomized controlled trials demonstrated that digital technology interventions significantly reduced maternal pain intensity (SMD = −1.49, 95% CI: −2.55 to −0.42, *p* = 0.006) and anxiety levels (SMD = −2.87, 95% CI: −4.37 to −1.36, *p* = 0.0002). Descriptive findings from two studies (20, 25) not included in the meta-analysis were consistent with the direction of these results, further supporting the beneficial effects of digital technology-assisted labor support. According to Cohen’s criteria, the pooled SMDs of −1.49 for pain and −2.87 for anxiety both represent large effects, suggesting substantial clinical relevance despite the absence of established minimal clinically important differences for these outcomes.

The findings of this study are consistent with previous primary research on the application of virtual reality for labor analgesia. Frey et al. (25) demonstrated in their crossover study that VR intervention significantly reduced sensory pain (difference −1.5), affective pain (difference −2.5), and cognitive pain (difference −3.1), which aligns completely with the direction of effect observed in our meta-analysis. Similarly, Carus et al. (20) reported that pain scores decreased significantly after VR use in early labor (2.6 ± 1.2 vs. 2.0 ± 1.3, *p* < 0.01), and overall childbirth satisfaction was significantly improved in the VR group. In recent years, several systematic reviews of non-pharmacological analgesic methods have also supported the findings of this study. A Cochrane systematic review by Smith et al. (34) included multiple studies on relaxation techniques (including music, audio analgesia, etc.) for labor pain management, concluding that these techniques could alleviate labor pain to some extent. The effect size observed in our meta-analysis (SMD = −1.49) suggests that digital technology-assisted labor support may have stronger analgesic effects than traditional non-pharmacological methods, which may be attributed to the multisensory immersive experience provided by VR technology that more effectively distracts parturients from pain signals (35).

The analgesic and anxiolytic effects of digital technology-assisted labor support, particularly immersive virtual reality, can be explained through multiple mechanisms. Firstly, the distraction theory represents the core mechanism: when the brain is occupied by multisensory information flow (visual, auditory, interactive experiences) from the virtual environment, neural resources available for processing pain signals are reduced, thereby decreasing pain perception (35). A functional magnetic resonance imaging study by Hoffman et al. (35) confirmed that activity in pain-related brain regions (including the insula, thalamus, and anterior cingulate cortex) was significantly reduced during VR intervention. Secondly, emotional regulation mechanisms also play an important role. The serene natural landscapes or fetal images provided by VR can induce positive emotions and reduce stress hormone levels (e.g., cortisol), thereby alleviating anxiety and tension (36). The study by Mohammadi et al. (28) showed that fear of childbirth scores were significantly lower in the VR group than controls at all stages of labor, suggesting that VR may indirectly influence pain experience through improved emotional states. Furthermore, enhanced self-efficacy represents another potential mechanism. By providing a sense of active participation and control (such as selecting game elements through eye movements in the Frey et al. (25) study), VR may enhance parturients’ confidence in coping with labor pain, which is closely associated with improved childbirth satisfaction (20).

Both outcomes in this meta-analysis—pain intensity and anxiety—showed high heterogeneity (*I*^2^ = 95 and 97%, respectively), warranting cautious interpretation. Potential sources of heterogeneity include: Differences in interventions: The digital technology content varied across included studies, including nature scene videos, 360° nature videos, seaside games, 4D fetal images, and intelligent management systems. The level of immersion and appeal may differ across different contents, potentially affecting intervention effects. It is important to acknowledge that the included interventions were not uniform. While we grouped them under the umbrella term “digital technology-assisted labor support,” the specific technologies varied: four studies employed immersive VR for pain distraction, one study used VR to display 4D fetal images, and one study implemented an intelligent delivery room management system. The latter differs mechanistically from VR-based distraction, as it primarily provides real-time monitoring and family-friendly environment to reduce anxiety. Nevertheless, only one study evaluated an AI/system-based intervention, precluding a separate meta-analysis for this category. The pooled effect estimates in our meta-analysis are therefore predominantly driven by VR-based studies. The high heterogeneity (*I*^2^ > 90%) reflects these clinical and methodological differences, and the overall SMDs should be interpreted as the average effect of a heterogeneous set of digital interventions, not as a uniform effect of a single technology. Differences in measurement time points: Pain and anxiety were measured at various time points, including the active phase of labor and the postpartum period. Pain intensity naturally increases with labor progression, and measurements at different time points may reflect different effect sizes. Differences in scale types: Anxiety was measured using four different scales (S-AI, Spielberger, PASS, Harman Fear of Childbirth Questionnaire), which differ in conceptual connotation and measurement range, potentially contributing to variation in effect sizes. Differences in population characteristics: Although the included studies predominantly enrolled primiparous women, differences in obstetric practices, cultural backgrounds, and pain perceptions across countries (Turkey, China, Iran, United States) may affect the generalizability of intervention effects. Sensitivity analysis showed that the pooled effect sizes remained stable after sequentially omitting each study, suggesting certain robustness of the results. However, the presence of heterogeneity indicates that future studies need to further standardize intervention protocols and measurement tools.

It is important to acknowledge that the observed analgesic and anxiolytic effects of VR may not be entirely attributable to the specific immersive experience. Placebo and contextual effects—such as the novelty of using a headset, heightened attention from care providers, the expectation of pain relief, and the sense of being cared for—are well-known modulators of pain perception. None of the included studies employed a sham VR condition (e.g., a headset displaying static images or non-immersive content) or an attention-matched control (e.g., a supportive companion providing equal duration of verbal distraction). Therefore, a portion of the observed effect sizes likely reflects non-specific factors rather than the specific mechanism of sensory distraction. Future randomized controlled trials should incorporate active control groups to isolate the specific contribution of immersion. Additionally, researchers are encouraged to measure expectancy and perceived support as covariates to better understand the relative contributions of specific and non-specific effects. Beyond these methodological considerations, translating these interventions into routine delivery room practice faces several practical challenges. First, hygiene and infection control: VR headsets contact the parturient’s face and may be contaminated by sweat, bodily fluids, or airborne particles. Establishing effective disinfection protocols without damaging the equipment is essential, especially in high-throughput delivery suites. Second, space and workflow integration: delivery rooms are often crowded with medical devices; storing, charging, and managing multiple headsets requires additional logistical support. Third, timing and patient cooperation: the optimal window for VR use appears to be cervical dilation of 4–6 cm, but unpredictable labor progression and intense contractions during active phase may limit a parturient’s ability to wear a headset or engage with VR content. Fourth, staff workload: midwives or nurses would need to assist with headset fitting, content selection, and troubleshooting, adding to their existing responsibilities. Fifth, cost and accessibility: although mobile phone-based headsets (e.g., Samsung Gear VR) cost as little as $50–100 per unit and standalone devices $300–500, these costs may still be prohibitive for low-resource settings. Moreover, recurring costs include content development, software updates, and device maintenance. Formal cost-effectiveness analyses have not yet been performed for VR-assisted labor support. We therefore recommend that future implementation research address these barriers and evaluate the technology’s affordability and acceptability in diverse healthcare systems.

Despite these caveats and implementation challenges, the findings of this study have important implications for obstetric clinical practice. Firstly, digital technology-assisted labor support, particularly VR interventions, can serve as an effective complement to traditional pharmacological analgesia, especially for healthy parturients who wish to minimize medical interventions. Secondly, the non-invasive nature and absence of side effects of VR technology confer a favorable safety profile, with no included studies reporting serious adverse events. Thirdly, with decreasing costs and improving portability of VR devices, this technology has good accessibility and potential for widespread implementation across various levels of maternity care facilities. Furthermore, digital technology-assisted labor support may not only improve pain and anxiety but also positively influence the overall childbirth experience. Carus et al. (20) reported significantly higher overall childbirth satisfaction in the VR group, with 95% of women expressing willingness to use VR in future labor. This has positive implications for promoting natural childbirth and reducing cesarean section rates. It is noteworthy that digital technology should be regarded as an adjunct rather than a replacement. In all included studies, parturients could receive routine obstetric care and pharmacological analgesia as needed, with VR intervention serving as a supplement rather than substitute, reflecting the “woman-centered” individualized care philosophy in modern obstetric practice (37).

This systematic review has several limitations. First, the number of included studies was small (*n* = 6), with only four studies eligible for meta-analysis, limiting the stability and generalizability of the results. Second, high heterogeneity was observed across studies; although sensitivity analyses were performed, the pooled effect sizes should be interpreted with caution. Third, all studies were unable to blind participants due to the nature of the intervention, potentially introducing performance bias. Fourth, most studies reported only short-term outcomes, lacking follow-up data on long-term postpartum psychological status (e.g., postpartum depression, post-traumatic stress disorder). Fifth, some studies lacked trial registration information, raising potential concerns for selective reporting bias. Sixth, due to the limited number of studies, publication bias assessment and subgroup analyses could not be performed. Seventh, the substantial clinical and technological heterogeneity across included interventions limits the interpretability of the pooled effect sizes as a single estimate for “digital technology” as a whole. In particular, the intelligent management system evaluated by Li et al. (26) differs mechanistically from the VR-based interventions. However, sensitivity analysis excluding this study confirmed the robustness of the overall findings, which are primarily driven by VR-based interventions. Future research should evaluate AI/system-based interventions separately once more studies become available.

Future research should focus on: conducting large-sample, multi-center, rigorously designed randomized controlled trials; standardizing intervention protocols and measurement tools to explore optimal VR content, duration, and frequency; extending follow-up periods to assess long-term effects on postpartum mental health; conducting cost-effectiveness analyses to evaluate the economic value of technology implementation in real-world settings; exploring differences in applicability of VR across cultural contexts and parity. Given the rapid evolution of digital technologies in obstetrics, an update of this systematic review will be warranted as new high-quality RCTs become available.

## Conclusion

This systematic review and meta-analysis demonstrates that digital technology-assisted labor support in delivery rooms, predominantly immersive virtual reality interventions, can significantly reduce maternal pain intensity and anxiety levels. Current evidence supports the use of digital technology as an adjunct to traditional pharmacological analgesia to improve the childbirth experience. However, due to the limited number of included studies and high heterogeneity, these conclusions require further validation through additional high-quality research. Future studies should focus on optimizing intervention protocols, extending follow-up periods, and exploring the applicability of this technology across different populations.

## Data Availability

The original contributions presented in the study are included in the article/Supplementary material, further inquiries can be directed to the corresponding author.

## Supplementary material

Appendix 1 Detailed search strategy.
