# Supplementary material for: Digital technology-assisted labor support in delivery rooms on maternal pain and anxiety: a systematic review and meta-analysis
Source: Front Med (Lausanne). 2026 Jul 22;13:1838637. doi: 10.3389/fmed.2026.1838637 (PMC13438421; doi:10.3389/fmed.2026.1838637)
Supplement: Supplementary file 1 [file Data_Sheet_1.DOCX]

PubMed (2026.3.4) (149):

(

"Virtual Reality"[Mesh] OR "Artificial Intelligence"[Mesh] OR "Telemedicine"[Mesh] OR "virtual reality"[tiab] OR "VR"[tiab] OR "artificial intelligence"[tiab] OR "AI"[tiab] OR "machine learning"[tiab] OR "mobile app"[tiab] OR "fetal monitoring"[tiab]

)

AND

(

"Labor, Obstetric"[Mesh] OR "Delivery, Obstetric"[Mesh] OR "labor"[tiab] OR "childbirth"[tiab] OR "delivery"[tiab]

)

AND

(

"Pain"[Mesh] OR "Anxiety"[Mesh] OR "pain"[tiab] OR "anxiety"[tiab] OR "fear"[tiab]

)

Embase (2026.3.4) (1397):

('virtual reality'/exp OR 'artificial intelligence'/exp OR 'telemedicine'/exp OR 'mobile application'/exp OR 'wearable computer'/exp OR 'fetus monitoring'/exp OR 'virtual reality':ti,ab OR 'vr':ti,ab OR 'artificial intelligence':ti,ab OR 'ai':ti,ab OR 'machine learning':ti,ab OR 'digital technology':ti,ab OR 'mobile app':ti,ab OR 'fetal monitoring':ti,ab)

AND

('labor'/exp OR 'obstetric delivery'/exp OR 'labor':ti,ab OR 'labour':ti,ab OR 'childbirth':ti,ab OR 'delivery':ti,ab OR 'intrapartum':ti,ab)

AND

('pain'/exp OR 'anxiety'/exp OR 'fear'/exp OR 'pain':ti,ab OR 'anxiety':ti,ab OR 'fear':ti,ab OR 'distress':ti,ab)

AND

('randomized controlled trial'/exp OR 'controlled clinical trial'/exp OR 'randomized controlled trial':ti,ab OR 'randomized':ti,ab OR 'randomised':ti,ab OR 'clinical trial':ti,ab OR 'systematic review'/exp OR 'meta analysis'/exp OR 'systematic review':ti,ab OR 'meta-analysis':ti,ab)

Cochrane Library (2026.3.5) (18):

(("virtual reality" OR VR OR "artificial intelligence" OR AI OR "machine learning" OR "fetal monitoring") AND (labor OR labour OR childbirth OR delivery OR intrapartum) AND (pain OR anxiety OR fear OR distress))

Web of Science (2026.3.5) (224):

((("virtual reality" OR VR OR "artificial intelligence" OR AI OR "machine learning" OR "fetal monitoring" OR "digital technology")) AND (labor OR labour OR childbirth OR delivery OR intrapartum) AND (pain OR anxiety OR fear OR distress))
